# Hydration properties and texture fingerprints of easy- and hard-to-cook bean varieties

**DOI:** 10.1002/fsn3.188

**Published:** 2014-12-07

**Authors:** Peter K Kinyanjui, Daniel M Njoroge, Anselimo O Makokha, Stefanie Christiaens, Daniel S Ndaka, Marc Hendrickx

**Affiliations:** 1 Department of Food Science and Technology, Faculty of Agriculture, Jomo Kenyatta University of Agriculture and TechnologyP.O. Box 62000-00200, Nairobi, Kenya; 2 Laboratory of Food Technology, Leuven Food Science Nutrition Research Center (LFoRCe), Department of Microbial and Molecular Systems (M^2^S), KU LeuvenKasteelpark Arenberg 22 Box 2457, 3001, Heverlee, Belgium

**Keywords:** Bean cooking, bean pretreatments, easy-to-cook, hard-to-cook defect, water-holding properties

## Abstract

The objective of this study was to understand the factors that affect the hydration and cooking profiles of different bean varieties. During this study, nine bean varieties were classified as either easy-to-cook (ETC) or hard-to-cook (HTC) based on a subjective finger pressing test and an objective cutting test. Rose coco, Red haricot, and Zebra beans were classified as ETC, while Canadian wonder, Soya fupi, Pinto, non-nodulating, Mwezi moja, Gwaku, and New mwezi moja were HTC. The effect of different soaking (pre)-treatments on the cooking behavior and/or water absorption of whole or dehulled beans was investigated. Dehulling, soaking in high pH and monovalent salt solutions reduced the cooking time of beans, while soaking in low pH and CaCl_2_ solutions increased the cooking time. Moisture uptake was faster in ETC and dehulled beans. Soaking at high temperatures also increased the hydration rate. The results point to pectin-related aspects and the rate of water uptake as possible factors that influence the cooking rate of beans.

## Introduction

Common dry beans (*Phaseolus vulgaris* L.) are one of the world's most important sources of human dietary protein (Pachio 1993; Leterme and Munoz 2002) and constitute an essential part of the diet for over 700 million people in the world (Leterme and Munoz 2002). Their consumption in Eastern and Southern Africa exceeds 50 kg per person per year (Wortmann and Kirkby 1998). In addition, they are good sources of carbohydrates, vitamins, and minerals, and are considered as a staple food in Sub-Saharan Africa (Taiwo et al. 1998). Production of common beans is characterized by a large variety of beans of different cooking properties. These cooking properties are dependent on several factors that include the seed size, variety, storage time, and conditions, precooking treatments as well as cooking methods (Uebersax et al. 1991; Nyakuni et al. 2008). Dry beans are, however, underutilized because of the long preparation times needed to achieve required palatability which raises the cost of their preparation (Vindiola et al. 1986; Uebersax et al. 1991).

Cooking time is one of the criteria used in evaluating beans cooking quality (Moscoso et al. 1984). Long cooking times are associated with the hard-to-cook (HTC) defect, a condition that aggravates during storage. Proneness of beans to the HTC defect has been determined to be a function of both variety and storage conditions (Giselle et al. 2004; Shiga et al. 2004). Several hypotheses to explain the development of the HTC defect have been proposed. Galiotou-Panayotou et al. (2007) developed the middle lamella-cation-phytate-phytase theory which suggests that pectates in the middle lamella are rendered insoluble upon cooking by replacement of their monovalent ions by calcium and magnesium ions. When the phytase hydrolyzes the phytate, the divalent cations released diffuse to middle lamella and combine with the pectates. Hincks and Stanley (1987) and Srisuma et al. (1989) on the other hand suggested that lignification occurs through the cross-linking of phenolics to cell wall proteins. Hincks and Stanley (1986), proposed a multiple mechanism of bean hardening which included phytate loss as a minor contributor during initial storage and phenol metabolism as a major contributor during extended storage. However, the complex mechanisms of the HTC phenomenon have not been clearly elucidated (Liu et al. 1992).

Cooking is intended to render beans palatable, digestible, and also to inactivate antinutritional factors (Taiwo et al. 1997). It is a hydrothermal process involving starch gelatinization, protein denaturation, and texture softening (Wang and Daun 2005). Prolonged cooking of beans due to the HTC defect has been reported to increase the percentage of leached solids, to destroy heat-labile vitamins and to decrease the protein quality of the cooked product (Walker and Kochhar 1982). Attempts to increase the utilization of legumes in general have employed a wide range of processing techniques such as soaking, boiling, autoclaving, radiation, cooking, roasting, dehulling, germination, fermentation, supplementation with various chemicals and enzymes and, recently, extrusion cooking (van der Poel 1990; Gujska and Khan 1991; Bishnoi and Khetarpaul 1994; Fernandez et al. 1997; Alonso et al. 1998, 2000).

Soaking dry beans forms an integral part of bean processing and its main purpose is to facilitate faster cooking (Abu-Ghannam and McKenna 1997). Soaking and cooking of beans are two separate processes that may or may not be performed simultaneously (Taiwo et al. 1997). Soaking, however, is time consuming requiring between 12 and 24 h at room temperature and therefore many attempts have been made to shorten it. The dehulling process has previously been applied to Sanilac and Pinto beans, and resulted in considerable reductions in soaking time. Increasing the temperature of the soaking solution has also been identified as another method for reducing the soaking time (Abu-Ghannam and McKenna 1997).

Since traditional cooking of dry edible beans in developing countries involves excessive expenditure of time and fuel, there is need to increase the acceptability and consumption of beans by tailoring existing and new processes to produce bean-based products that address this problem. This requires innovative scientific and technological approaches for changing the physicochemical properties of beans to meet certain functionalities. This study aimed to classify some common dry bean varieties as either easy-to-cook (ETC) or HTC. The effect of different soaking (pre)-treatments and dehulling on the cooking behavior of the two classes of beans was also investigated.

## Materials and Methods

### Beans samples

Nine varieties of beans (*P. vulgaris* L.) were purchased from Kenya Agricultural Research Institute (KARI), Thika Station. They were from different harvesting seasons giving rise to 16 samples. After harvesting, the beans were solar dried to a moisture content of 10%, dusted with pesticides and stored under controlled relative humidity and temperature at KARI research station in Thika. The beans had been stored for between 1 and 15 months under controlled temperature and humidity conditions. These included Rose coco (TKA 13, TKA 15 and GLP 2), Red haricot (TKA16, GLP 15 and GLP 585), Zebra beans (TKA 22), Canadian wonder (GLP 24, TKA 17 and TKA 23), Soya fupi (TKA 19), Pinto (GLP X92), non-nodulating (TKA 19), Mwezi moja (GLP 1004), Gwaku (TKA 21), and New mwezi moja (GLP 1127a) common beans. These bean varieties were selected since they are among the most popular bean varieties in Kenya and had all been bred and grown at KARI Thika station under similar and controlled agronomic conditions. The beans were used as such (whole) or manually dehulled.

### Classification of beans as ETC or HTC

#### Cooking of beans

One hundred unsoaked seeds from each of the nine varieties of common beans were subjected to standard cooking at 96°C in a thermostated water bath (WBU-45; Memmert, Schwabach, Germany) for 30 min intervals. The cooked samples were withdrawn and cooled in a cold water bath for 1 min before determining the cooking status. Beans which took 2 h or less for 80% of the seeds to cook at 96°C were classified as ETC, else they were considered to be HTC. This is based on the traditional practice where beans cooking within 2 h are considered ETC.

#### Finger pressing test

The softness/hardness (cookability) of the beans was determined subjectively by pressing the cooked beans between the thumb and forefinger (Vindiola et al. 1986). The beans were classified as cooked when the cotyledons disintegrated on pressing. The percentage of cooked beans in the batch was determined as a function of time.

#### Cutting test

The softness/hardness (cookability) of the beans was also determined objectively using a Sun-Rheometer (Compact 100 Model CR-100; Sun Scientific Company Ltd, Tokyo, Japan) equipped with a Graphtec (Yokohama, Japan) (Servo 150 SR 6511) recorder. The system uses a cutting probe which could measure up to a maximum force of 100 N at a speed of 100 mm min^−1^. The maximum force registered during the cutting of the cooked beans was recorded. Every experimental value indicates the average of 10 measurements for a given time. The data obtained were used to generate cooking curves for the different varieties. To convert the cutting test data to percent cooked beans, the average cutting force of the fully cooked beans was determined for the different bean varieties. This force (11.16 ± 3.94 N) was then used to discriminate between the cooked and uncooked beans. Subsequently, the data obtained were used to generate cooking curves for the different varieties.

### Soaking of beans in different solutions

Beans were soaked in different solutions to determine their subsequent cooking behavior according to the modified method of Clemente et al. (1998). The solutions tested included deionized water, 0.1 mol/L monovalent (NaCl, NaHCO_3_, Na_2_CO_3_) and 0.1 mol/L divalent (CaCl_2_) salts, and solutions of varying pH (4, 5, 6, 8, 8.5). Acetate buffer was used at the low pH range (4, 5 and 6) while phosphate buffers were used at high pH (8 and 8.5). Soaking was carried out in each of the brine solutions at 25°C for 6 h followed by cooking at 96°C for 30, 60, and 90 min. The cooking profile of an ETC variety (Zebra beans) and a HTC variety (Soya fupi beans) with or without soaking in the varying solutions was investigated.

### Determination of water absorption

#### Moisture content

Initial moisture content of the beans was determined according to the AOAC method 930.15 (Association of Official Analytical Chemists (AOAC) 2000). The beans samples were ground into fine flour. About 5 g of each ground sample was accurately weighed into a moisture dish and transferred in to a hot-air oven at 103°C and then dried until a constant weight was attained. The final weight of the samples was taken after cooling them in desiccators. The residue was taken as the total solids and loss in weight as the moisture content of the sample.

#### Water absorption during soaking

Water absorption in both whole and dehulled bean samples during soaking was determined by placing a sample of approximately 10 g (weighed exactly) in a 50 mL beaker containing 40 mL of deionised water heated to the required soaking temperature (20, 35, and 50°C). The beakers were placed in different water baths (Memmert WBU-45) thermostatically controlled at the required temperature according to the modified procedures of Turhan et al. (2002) and Sayar et al. (2001). Water absorption was recorded every 30 min up to a total soaking time of 360 min for whole beans and 300 min for dehulled samples. As the rate of water absorption declined considerably approaching equilibrium, monitoring times were extended to 1 h towards the end of soaking. At the different soaking times, the beans were removed from the soaking solution, drained for 2 min, blotted with tissue paper and weighed. The weight gain was calculated and the beans were returned to the soaking solution at the defined temperature. All soaking tests were carried out in triplicate and average results were used to calculate the percentage moisture gain (Abu-Ghannam and McKenna 1997).

#### Water absorption during cooking

Water absorption of both whole and dehulled beans samples during cooking was determined by placing a sample of approximately 10 g (weighed exactly) in 50 mL pyrex cooking tubes containing 40 mL of deionised water preheated to 96°C. The tubes were placed in a thermostatically controlled water bath at 96°C and the temperature was maintained constant throughout the entire cooking experiment. Water absorption was recorded every 15 min up to the point where all the beans were fully cooked. After specified cooking times, the beans were removed from the cooking solution, drained for 2 min, blotted with tissue paper and weighed (Sayar et al. 2001 and Turhan et al. 2002). The process was continued until all the beans in the tube were cooked (100% cooked). All cooking tests were carried out in triplicate and average results were noted as percentage moisture gain.

### Data analysis

Statistical analysis was performed on data collected from the soaking and cooking tests. The mean values of the different bean varieties were compared using the least square difference (LSD) at the 5% significance level.

## Results and Discussion

### Classification of different bean varieties as ETC or HTC

**Figure 1:**
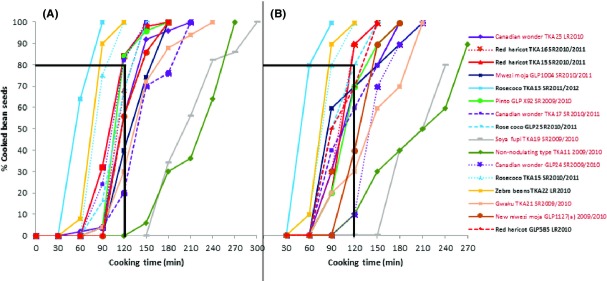
Cooking profiles of various common bean varieties (*Phaseolus vulgaris*). (A) based on the (subjective) finger pressing test and (B) based on the (objective) cutting test where an average cutting force of 11.16 ± 3.94 N was used to discriminate between cooked and uncooked beans. The bold black lines indicate the boundary between ETC and HTC beans. Beans taking 120 min or less for 80% of the seeds to be cooked at 96°C are classified as ETC, otherwise HTC. Varieties marked in black in the figure legend are ETC and varieties marked in red in the figure legend are HTC.

The cooking profiles of the different bean varieties as determined by finger pressing (subjective method) and by cutting test (objective) are shown in Figure1. The average cutting force of the cooked beans was determined (11.16 ± 3.94 N) and used to discriminate between cooked and uncooked beans and the results used to generate cooking profiles based on the objective determination of cooked beans as shown in Figure1B. The two methods resulted in a similar categorization of the beans into ETC and HTC clusters. Rose coco (TKA 13, TKA 15 and GLP 2), Red haricot (TKA16, GLP 15 and GLP 585), and Zebra beans (TKA 22) were classified as ETC whereas Canadian wonder (GLP 24, TKA 17 and TKA 23), Soya fupi (TKA 19), Pinto (GLP X92), non-nodulating (TKA 19), and Mwezi moja (GLP 1004, GLP 1127a and TKA 21) beans were considered to be HTC. The similarity of the resulting curves of the two methods show that if practiced by experienced investigators, the finger pressing method is as accurate as the objective method for determining cooking rates. Some earlier studies (Bourne 1982; Yeung et al. 2009) have advocated for sensory analysis (including finger pressing) in beans cooking quality determination due to their greater sensitivity and lower cost of application. It was also observed that the cooking time of beans was influenced by the duration of storage for the same variety of beans. For example, Rose coco harvested in the short rains of 2011/2012 cooked faster than Rose coco harvested in the short rains of 2010/2011, although they were both within the cluster of ETC beans. Similar results were observed for the Canadian wonder beans from seasons 2009/2010 and 2010/2011, although they both fell within the HTC cluster. This is in line with the findings of Nyakuni et al. (2008). Finally, the cooking curves appeared to be sigmoid in nature showing an initial lag phase and a subsequent exponential phase followed by a final slowing down in the cooking rate of the beans as the curves neared 100% cooking. The lag and exponential phases seemed to be influenced by both the variety and storage time.

### Effect of different soaking solutions on the cooking behavior of ETC and HTC beans

**Figure 2:**
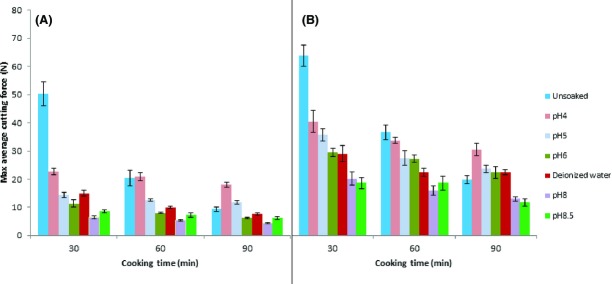
Effect of soaking ETC and HTC beans in solutions of varying pH followed by cooking at 96°C for varying time intervals on the hardness of the beans. (A) Zebra beans (ETC) (B) Soya fupi beans (HTC).

Figure2 shows the cooking behavior of different bean varieties after soaking the beans in different solutions. Both ETC (Zebra) and HTC (Soya fupi) beans soaked in deionized water, the cooking rates were faster than the unsoaked beans. The beans soaked in low pH had a slower cooking rate than the beans soaked in deionized water, while the beans soaked under high pH cooked faster. It was also observed that the lower the pH, the slower the cooking rate within the limits of the experiment (which was pH 4). At high pH, the cooking rate was faster. Potter (1973) suggested that alkaline solutions (high pH) act as tenderizers and cause breakdown of proteins, polysaccharides, and pectin substances. These changes increase cell permeability thus increasing the cooking rate. Sila et al. (2006) suggested that high temperatures and alkaline pH favor *β*-elimination of pectin thus making the cells easier to separate during cooking thus reducing the cooking time. Haladjian et al. (2003) also observed that beans soaked in alkaline (high) pH exhibited higher hydration rates which coupled with the *β*-elimination of pectin increases the cooking rate. The higher hydration rates could possibly be attributed to increased cell membrane permeability (Wanjekeche et al. 2003). This behavior is thus in line with the hypothesis that pectin is involved in the development of the HTC defect in beans.

**Figure 3:**
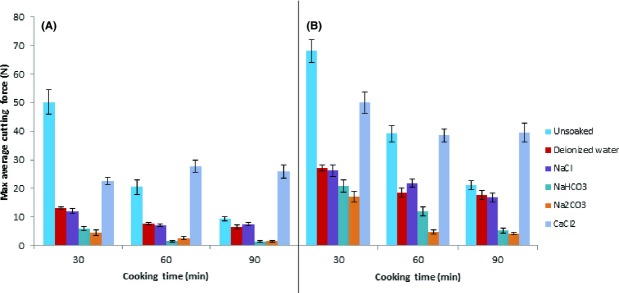
Effect of soaking ETC and HTC beans in different solutions followed by cooking at 96°C at varying time intervals on the hardness of the beans. (A) Zebra beans (ETC), (B) Soya fupi beans (HTC).

Monovalent salts, in particular Na_2_CO_3_ and NaHCO_3,_ had a pronounced effect on increasing the cookability of both ETC and HTC beans (Fig.3). The cooking effect was larger with increasing cooking time unlike for the beans soaked in high pH solution which shows that although their action could be partially attributed to their alkalinity, part of their activity seems to be specific to these type of salts. Conversely, CaCl_2_ had a hardening effect on both ETC and HTC beans. This observation can be attributed to cross-linking of pectin with calcium ions to form insoluble pectates which render the cells resistant to water absorption and the subsequent failure of adjacent cells to separate upon cooking as suggested by Bressani 1993 and Kilmer et al. (1994). As the cooking time increased from 30 to 90 min, the effect of CaCl_2_ is manifested more clearly, where after 90 min maximum average cutting force for the CaCl_2_ soaked beans is relatively much higher than the cutting force for all the other treatments, including the unsoaked beans. This hardening effect is in line with the HTC defect which manifests during cooking since it prevents the separation of cells in the cooked food materials (Nyakuni et al. 2008). These findings are also in line with the findings of Onwuka and Okala (2003) who showed that adding calcium to the cooking water significantly increases the cooking time. NaCl on the other hand had no effect on the cooking rates of both ETC and HTC bean varieties. This is contrary to the findings of Bertolodo et al. (2009) who showed that addition of NaCl in soaking solutions significantly decreased the cooking time of beans. Deducing from the effects of the monovalent and divalent ions on the cooking behavior of the beans, a postulation that pectin plays a role in the development of the HTC defect in the beans is developed.

### Water absorption during soaking and cooking of ETC and HTC beans

**Figure 4:**
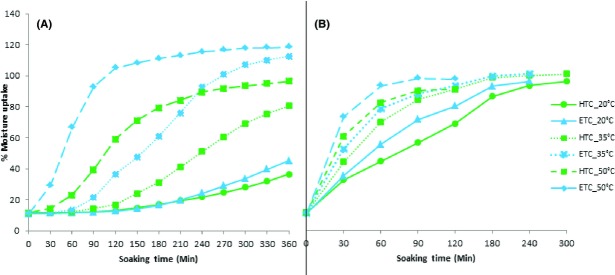
Percent moisture uptake as a function of soaking time and temperature for ETC beans (Rose coco) and HTC (Pinto). (A) whole beans, (B) dehulled beans.

The water absorption behavior during soaking of ETC (Rose coco) and HTC (Pinto) beans is shown in Figure4. The amount of moisture absorbed increased with increasing soaking times. The whole beans profiles (Fig.4A) were characterized by an initial lag phase followed by an exponential phase and lastly a plateau phase which presumably occurred as water filled up all the free capillary and intermolecular spaces (Sopade et al. 1992). The rate of water absorption also increased with increasing temperatures as the temperature rose from 20 to 35°C and finally to 50°C. Furthermore, the ETC bean variety (Rose coco) absorbed water faster than the HTC bean variety (Pinto) at all temperatures especially when soaking whole beans. This could be one of the possible reasons why the ETC beans cook faster since during cooking at temperatures above gelatinization temperature, starch is immediately gelatinized in presence of sufficient moisture (ratio of 1:0.75) (Engels et al. 1986; Hoseney 1994; Turhan and Gunesakaran 2002) which therefore means that the faster the moisture uptake, the higher the cooking rate of beans.

In the dehulled beans (Fig.4B), the lag phase was completely absent and the beans immediately started taking up water at an exponential rate even at the lower soaking temperatures. This observation implies that the seed coat is one of the rate-limiting factors in beans cooking. This is similar to the findings of Sayar et al. 2001. The varietal differences were still very clear even with dehulling with the ETC beans absorbing moisture faster than the HTC beans at the different soaking temperatures. At 20°C, both the dehulled ETC and HTC had attained a moisture content of above 100% after 300 min while without dehulling none of the two varieties had attained 40% moisture content (Rose coco at 39.8% and Pinto at 32.5%).

**Figure 5:**
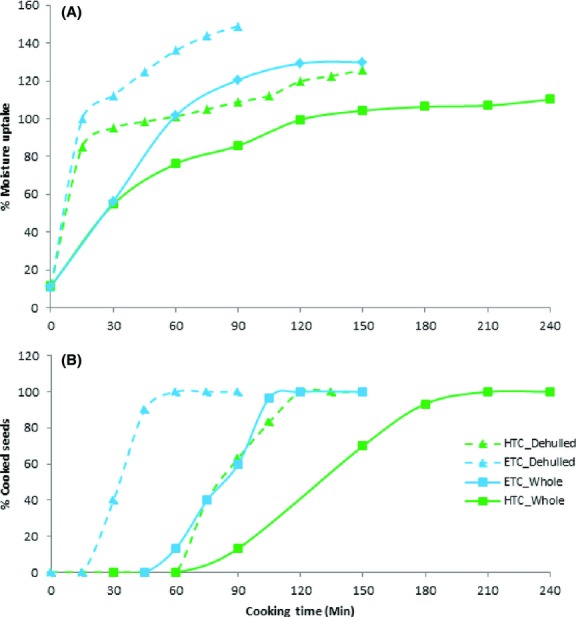
Cooking behavior of whole and dehulled ETC beans (Rose coco) and HTC (Pinto) as a function of cooking time. (A) Percent moisture uptake (B) Percentage cooked beans.

As during soaking, during cooking, the ETC beans absorbed moisture faster than the HTC beans. They also absorbed more moisture (Fig.5). This could partly explain the faster cooking rate of the ETC beans. Dehulling also increased the rate of moisture uptake during cooking for the two bean varieties. This shows that dehulled seeds required a shorter activation period to allow water to diffuse in the seeds. The ETC (Rose coco) beans took 90 min while the HTC (Pinto) whole beans took 240 min to be fully cooked (Fig.5). Dehulling reduced the cooking time of the ETC beans from 90 to 45 min (50%) and the HTC beans from 240 to 105 min (56%). It has been hypothesized that the seed coat forms a barrier to moisture penetration thus reducing the rate of starch gelatinization as well as pectin solubilization of cotyledon cells which leads to beans softening (Silva et al. 1981; Clemente et al. 1998).

## Conclusions

In order to save on the cooking time and fuel, common bean consumers in Kenya can adopt the three beans varieties classified as ETC (Rose coco, Red haricot and Zebra) since they cook faster. To further improve on the cooking time, they can dehull or soak the beans in either high pH or monovalent salt solutions. Soaking in CaCl_2_ and low pH solutions tended to increase the firmness of the cooked beans and thus prolonged the cooking time and can thus be adopted by bean canners especially in bean varieties that easily mush during canning. The mechanism responsible for the prolonged cooking time in common beans is complex. Results from this study point at both pectin and the seed coat as possible influencers of beans' cooking time.

## References

[b1] Abu-Ghannam N, McKenna B (1997). Hydration kinetics of red kidney beans (*Phaseolus vulgaris L*.). J. Food Sci.

[b2] Alonso R, Orue E, Marzo F (1998). Effects of extrusion and conventional processing methods on protein and anti-nutritional factor contents in pea seeds. Food Chem.

[b3] Alonso R, Aguirre A, Marzo F (2000). Effect of extrusion and traditional processing methods on anti-nutrients and in vitro digestibility of protein and starch in faba and kidney beans. Food Chem.

[b4] Association of Official Analytical Chemists (AOAC) (2000). AOAC official methods of analysis.

[b5] Bertolodo JG, Rocha F, Barili LD, Vale NM, Coimbra JLM, Guidolin AF (2009). Salts concentrations in combination with soaking time: effect in bean cooking time. Ciência e Tecnologia de Alimentos. Campinas.

[b6] Bishnoi S, Khetarpaul N (1994). Saponin content and trypsin inhibitor of pea cultivars: effect of domestic processing and cooking methods. J. Food Sci. Tech.

[b7] Bourne MC (1982). Food texture and viscosity: concept and measurement.

[b8] Bressani R (1993). Grain quality of common beans. Food Rev. Int.

[b9] Clemente A, Sanchez-Vioque R, Vioque J, Bautista J, Milla F (1998). Effect of processing on water absorption and softening kinetics in chickpea (*Cicerarietinum* L) seeds. J. Sci. Food Agric.

[b10] Engels C, Hendrickx M, de Samblanx S, de Gryze I, Tobback P (1986). Modeling water diffusion during long-grain rice soaking. J. Food Eng.

[b11] Fernandez M, Aranda P, Lopez-Jurado M, Garcia-Fuentes MA, Urbano G (1997). Bioavailability of phytic acid phosphorus in processed *Vicia faba* L. var. Major. J Agr. Food Chem.

[b12] Galiotou-Panayotou M, Kyriakidis NB, Margaris I (2007). Phytase-phytate-pectin hypothesis and quality of legumes cooked in calcium solutions. J. Sci. Food Agric.

[b13] Giselle AM, Banu FO, Lisa JM, Nielsen SS (2004). Analysis of hard-to-cook Red and Black common beans using Fourier transform infrared spectroscopy. J Agr. Food Chem.

[b14] Gujska E, Khan K (1991). Feed moisture effects on functional properties, trypsin inhibitor and hemagglutinating activities of extruded bean high starch fractions. J. Food Sci.

[b15] Haladjian N, Fatad R, Toufeili I, Shadarevian S, Sidahmen M, Bayduon E (2003). pH, Temperature and Hydration kinetics of Faba beans (*Vicia faba* L). J Food Process Pres.

[b17] Hincks MJ, Stanley DW (1986). Multiple mechanisms of bean hardening. J. Food Technol.

[b18] Hincks MJ, Stanley DW (1987). Lignification: evidence for a role in hard-to-cook beans. J. Food Biochem.

[b19] Hoseney RC (1994). Principles of cereal science and technology.

[b21] Kilmer OL, Seib PA, Hoseney RC (1994). Effects of minerals and apparent phytase activity in the development of hard-to-cook state of beans. Cereal. Chem.

[b22] Leterme P, Munoz LC (2002). Factors influencing pulse consumption in Latin America. Br. J. Nutr.

[b23] Liu K, Phillips RD, Hung YC, Shewfelt RL, Mcwatters KH (1992). Hard-cook-defect in cowpeas: storage-induced and treatment induced development. J. Food Sci.

[b24] Moscoso W, Bourne MC, Hood LF (1984). Relationships between the hard-to-cook phenomenon in red kidney beans and water absorption, puncture force, pectin, phytic acid, and minerals. J. Food Sci.

[b25] Nyakuni GA, Kikafunda JK, Muyonga JH, Kyamuhangire WM, Nakimbugwe D, Ugen M (2008). Chemical and nutritional changes associated with the development of the hard-to-cook defect in common beans. Int. J. Food Sci. Nutr.

[b26] Onwuka UN, Okala O (2003). Effects of selected salts on cooking time, protein content and sensory properties of African yam beans and cowpeas. Food Serv. Tech.

[b27] Pachio D, Henry G (1993). The demand for bean technology. Trends in CIAT commodities.

[b28] van der Poel AF (1990). Effect of processing on antinutritional factors and protein nutritional value of dry beans (*Phaseolus vulgaris* L.). Anim. Feed Sci. Tech.

[b29] Potter NN (1973). Food Science.

[b30] Sayar S, Turhan M, Gunasekaran S (2001). Analysis of chickpea soaking by simultaneous water transfer and starch water reaction. J. Food Eng.

[b31] Shiga TM, Lajolo FM, Filisetti CC (2004). Changes in the cell wall polysaccharides during storage and hardening of beans. Food Chem.

[b32] Sila DN, Smout C, Elliot F, Van Loey A, Hendrieckx M (2006). Non-enzymatic depolymerization of carrot pectin: towards a better understanding of carrot texture during thermal processing. J. Food Sci.

[b33] Silva C, Bates R, Deng J (1981). Influence of soaking and cooking upon the softening and eating quality of black beans (*Phaseolus vulgaris* L). J. Food Sci.

[b34] Sopade PA, Ajisegiri ES, Badau MH (1992). The use of Peleg's equation to model water absorption in some cereal grains during soaking. J. Food Eng.

[b35] Srisuma N, Hammerschmidt R, Uebersax MA, Ruengsakulrach S, Bennink MR, Hosfield GL (1989). Storage induced changes of phenolic acids and the development of hard-to-cook defect in dry beans (*Phaseolus vulgaris* var. Seafarer). J. Food Sci.

[b36] Taiwo KA, Akanbi C, Ajibola OO (1997). The effects of soaking and cooking time on the cooking properties of two cowpeas varieties. J. Food Eng.

[b37] Taiwo KA, Akanbi CT, Ajibola OO (1998). Regression relationships for the soaking and cooking properties of two cowpea varieties. J. Food Eng.

[b38] Turhan M, Gunesakaran S (2002). Kinetics of in situ and in vitro gelatinization of hard and soft wheat starches during cooking in water. J. Food Eng.

[b39] Turhan M, Sayar S, Gunasekaran S (2002). Application of Peleg model to study water absorption in chickpea during soaking. J. Food Eng.

[b40] Uebersax MA, Ruengsakulrach S, Occea LG (1991). Strategies and procedures for processing dry beans. J. Food Tech.

[b41] Vindiola OL, Seib OA, Hoseney RC (1986). Accelerated development of hard-to-cook state in beans. Cereal Foods World.

[b42] Walker AF, Kochhar N (1982). Effect of processing including domestic cooking on nutritional quality of legumes. Proc. Nutr. Soc.

[b43] Wang N, Daun JK (2005). Determination of cooking times of pulses using an automated Mattson cooker apparatus. J. Sci. Food Agric.

[b44] Wanjekeche E, Wakasa V, Mureithi JG (2003). Effect of germination, alkaline and acid soaking and boiling on the nutritional value of mature and immature mucuna (*Mucuna pruriens*) beans. Trop. Subtrop. Agroecosyst.

[b45] Wortmann C, Kirkby R (1998).

[b46] Yeung H, Ehlers JD, Waniska RD, Alviola JN, Rooney LW (2009). Rapid screening methods to evaluate cowpea cooking characteristics. Field Crops Res.

